# Investigation on the Influence of Microstructure Based on Hydrogen Bonding on Surface Tension by Raman Spectroscopy

**DOI:** 10.1155/2019/7975237

**Published:** 2019-12-29

**Authors:** Nannan Wu, Shunli Ouyang, Junjie Cui, Shiliang Liu, Mingzhe Zhang, Qingcheng Hu, Baokun Huang

**Affiliations:** ^1^Key Laboratory of Integrated Exploitation of Bayan Obo Multi-Metal Resources, Inner Mongolia University of Science and Technology, Baotou 014010, China; ^2^College of Science, Inner Mongolia University of Science and Technology, Baotou 014010, China; ^3^School of Energy and Environment, Inner Mongolia University of Science and Technology, Baotou 014010, China; ^4^Electronic Engineering School, Huaihai Institute of Technology, Lianyungang 222000, China

## Abstract

Surface tension and Raman spectra containing hydrogen bonding in acetonitrile aqueous solutions with different mole ratios were obtained. Varied surface tension and hydrogen bonding in the mixed solution were discussed. For this purpose, the OH stretching bands were fitted into three Gaussian components to which different hydrogen-bonded water samples were assigned. Furthermore, the microstructures of binary solution were analyzed. The results indicated that the surface tension decreases dramatically with the enhancement of hydrogen bonds in the mixture. A spectroscopic method for studying the macroscopic properties of aqueous solutions was employed. The direct experiment results provided the relationship between surface tension and microstructure in aqueous solutions.

## 1. Introduction

Water, as the most common solvent, is widely used in the chemistry, biology, medicine, and life sciences [1–5]. So far, it was found that water is the simple substance with peculiar physical and chemical properties, the density in solid water was brought being smaller than it in liquid water [5]. It is the change of water substructure that distinguished from the metallic and nonmetallic materials due to the change of water substructure from the structure of chain and/or cyclic annular to a stable three-dimensional network structure in the transformation of state through hydrogen-bonding interaction. It is the reason for the nature being full of life rather than a frozen world. Indeed, the hydrogen bond structure was considered as the basis of water structure and the changes in it would lead to abnormal physical and chemical properties of aqueous solutions that attributed to the microstructure of water based on hydrogen-bonding networks [1, 6–8]. Apparently, the transformation of various hydrogen bond modes obtained by molecular dynamic simulation in water was related to the anomalous physical properties of water near 4°C [9]. Afterwards, varied trend in dielectric constant and boiling point in contrast to hydrogen-bonding and nonhydrogen-bonding solutions was observed [10], indicating that the solution with hydrogen bond action has an abnormal dielectric constant and the increasing boiling point of the substance led the average up to 76°C. The common view on the structure model of liquid water is shown, mainly by having the tetrahedral structure. It was similar to the ice with a complex structure [11]. The interaction of hydrogen bonds in mixture increased the solubility of the solute [12, 13]. The results indicated that the DMSO aqueous solution with the antifreeze effect was better than pure DMSO solutions [14]. Surface tension is a kind of physical phenomenon that is limited to the special stress state between thin layers of liquid surface, which is a representation of intermolecular force. It is the physical cause of bubbles, capillary phenomenon, and concave and convex liquid levels in daily life. In the petrochemical industry, pipeline transportation, surfactant, sewage treatment, and other production practices are of great concern. It is of great significance to study the effect of intermolecular microinteraction on surface tension for understanding surface tension and its applications. Meantime, it made us take intense interest because macroscopic properties were affected by the microcosmic interaction of hydrogen bonds in aqueous solutions.

Hydrogen bonding, a kind of special weak intermolecular force, has the characteristics of directional attractions with saturation [1, 7]. Meantime, covalent bonding was exhibited, depending on the combination of hydrogen atoms in the molecule with the atoms of X possessing the large electronegativity and small radius. X-H-Y-type intermolecular and molecular interaction linked by the hydrogen atom was formed as X was close to the other counterpart Y. Much attention on the hydrogen bonds was paid after being proposed firstly by Huggins in 1920 and then elaborated by Linus Pauling in the book “Nature of Chemical Bonds” [15]. Hydrogen bonding is extensively present in the solution system. Microheterogeneity and the microstructure of the mixture were affected by the intramolecular and intermolecular hydrogen-bonding interaction [16, 17]. Meanwhile, hydrogen bonding played a vital role in the structure of proteins and the stability of DNA double helix structures [5, 18, 19]. The intermolecular hydrogen bonding increased the melting point and boiling point of the substance [20–22]. Wang et al. [20] found that the influence of the crystal structure and molecular structure of azolol explosives on the melting point using the nidazole compound was studied. It was found that the molecular structure was changed by the hydrogen bond, and the melting point of the explosive increased. Importantly, the melting point could be reduced by replacing the H atom with the suitable substituent. Otherwise, hydrogen bonding increased the viscosity of polyhydroxy compounds. For example, the effect of hydrogen bonding between polymer chains and water molecules on the viscosity of polyvinyl alcohol aqueous solution was seen [23]. As a result, the rheological properties of the solution depend on the relative strength of hydrogen bonds. When the temperature, pressure, or degree of hydrolysis changed, the hydrogen bond structure would break, making the viscosity decrease. Hence, the association was observed in the liquid molecules with the formation of hydrogen bonds, by which the density of solution was affected. Our concern has been aroused by the surface tension of solution, owing to the influence of hydrogen bonding on many macroscopic properties. A binary system of acetonitrile aqueous solution with strong hydrogen bonding was selected to explore the reason of surface tension resulting from hydrogen bonding.

Raman spectroscopy, as a scattering spectroscopy technique, with its lossless and noncontact in situ detection advantage, is extensively applied in food safety, biology, chemistry, medicine, and material science. Up to now, Raman spectroscopy, which was an important technical approach, has been applied in a lot of work to study the structure of solutions [14, 24, 25], especially affiliating the great improvement in the structure and weak interaction of the solution system. It can be used to know the interaction between molecules and the molecular role, changes in the process of chemical reaction, the nature of solution, and so on. The OH vibrational peak in the range of 2800–4000 cm^−1^ is the signal region reflecting the water structure [24–29]. Nevertheless, much controversy is not exactly known. Gauss fitting, a very excellent method, is often used to understand the vibrational modes in Raman spectra. The OH stretch band can often be fitted with five [24, 25, 30–33], four [34, 35], three [36], and two [37, 38] Gaussian components, and different structure models could also be further attributed to each subbond. Nowadays, there are two classical models that are accepted mostly for investigating the vibrational peaks of the OH stretching vibration band, namely, vibration models [37] (symmetric stretching vibration and antisymmetric stretching vibration) and structural models [24, 25, 39] (free water, partially hydrogen-bonded structure, and fully hydrogen-bonded structure). In order to explore the influence of structure on the properties of solutions, these models were provided to know the structure of aqueous solutions. The final purpose of application on the water was attributed to the investigation of its complex structure and prevention of the microscopic changes on macroscopic properties. Therefore, it is an important issue for establishing its relationship between the macroscopic/microscopic properties of liquid water and the microstructure of liquid water in the field of water science [11].

In this work, a Gauss deconvolution method was used to fit the Raman peak of the OH bond in the acetonitrile-water binary aqueous solution, and the change in the solution structure resulting from the interaction in the mixture and surface tension was discussed. An attempt which is relevant to the basic experimental data for studying the relationship between microstructure and macroscopic properties of aqueous solutions can be taken.

## 2. Materials and Methods

### 2.1. Reagents and Preparation

Acetonitrile used in the experiment is in purity greater than 99.9%. Ultrapure water (HPLC grade; resistivity: 18.2 MΩ·cm) was purchased from J&K Scientific Ltd. (Beijing, China). All the reagents were used without further purification. Acetonitrile-water mixed solution was prepared in different mole ratios (nCH_3_CN : nH_2_O) at 1 : 20, 1 : 10, 1 : 6, 1 : 5, 1 : 4, 1 : 3, 1 : 2, 3 : 4, 1 : 1, 3 : 2, 2 : 1, 5 : 2, 3 : 1, 4 : 1, 5 : 1, 6 : 1, 10 : 1, and 20 : 1. All tested solutions were mixed adequately.

### 2.2. Experimental Apparatus

The surface tension was measured [40] by using a ZL-10-type automatic digital display interface tensiometer purchased at Zibo Aiji Electric Co., Ltd., (Shandong, China), with the measuring range at 2 to 200 mN/m; accuracy and resolution are 0.1 mN/m and 0.2 mN/m, respectively.

The Raman spectrum system [40] is constructed independently by our laboratory equipped with an Andor Shamrock SR-500i-C-R-type spectrometer and Andor iDus CCD (charge-coupled device) detector produced by the UK ANDOR company, and a 1200 grove/mm grating with a wavelength resolution of about 0.05 nm was employed. 50 times long focus objective lens (parameter: 50×/0.35) was used to focus the samples. The focal point was completed by adjusting the sample height, and the focused images were collected by using the SunTime130E CMOS color digital camera and excited at the 532 nm line of semiconductor laser whose output power is ∼25 mW at each sample. And then, a single-crystal silicon prototype was calibrated using the Raman spectrometer ranging from 0 to 4000 cm^−1^ for scanning. The signal data of each sample was gathered at room temperature with 1.5 seconds exposure time, 2 times accumulation number, and 10 s accumulation cycle time.

## 3. Results and Discussion

**Figure 1:**
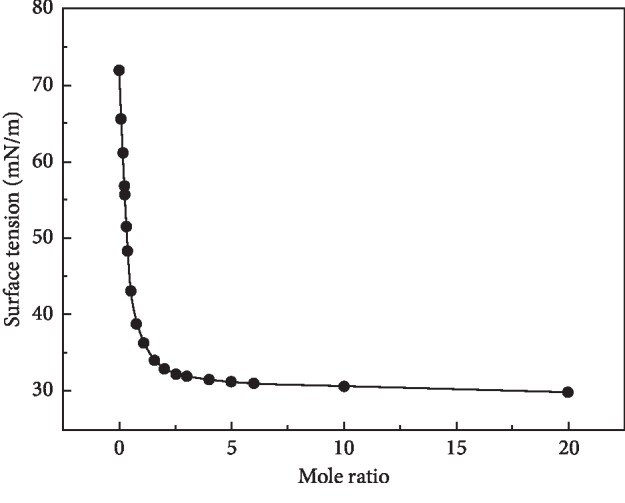
Relationship between surface tension and mole ratio of acetonitrile aqueous solution.

**Figure 2:**
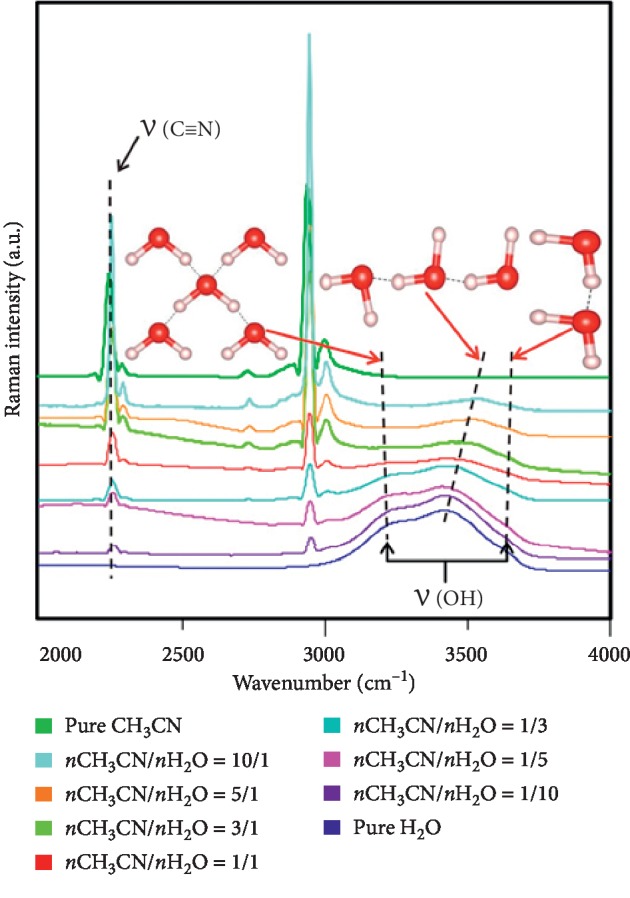
Raman spectra of pure acetonitrile, pure water, and acetonitrile aqueous solution with different mole ratios.

In the experiment, the surface tension of all mixtures, neat acetonitrile, and water was measured multiple times. Taking the average of multiple measurements, the surface tension of acetonitrile and water at 27.9 and 72 mN/m, respectively, which is similar to the experiment and theoretical calculation results given by Pátek [41], obtained the relationship between the surface tension and the mole ratios, as shown in Figure 1. The Raman spectrum including the interaction signal between acetonitrile and water of different solutions was recorded, and the microcosmic interaction was investigated by the changing trend graph of the CN stretching bond and OH stretching bond with mole ratio in different solutions, as shown in Figure 2.

Figure 1 depicts the relative curve of surface tension of acetonitrile aqueous solution with concentration. With the raise in acetonitrile content, the solution surface tension decreases significantly as the mole ratio is less than 1 : 1. However, the variation in surface tension is sluggish, indicating the small tension gradient was formed as the mole ratio is greater than 1 : 1. The experimental results of investigated specimens show that the change in surface tension in the mixture is limited by two pure solutions, being related to the content of water and acetonitrile, mainly by the two kinds of pure solutions determined when the mixture is close to each neat solution. There are two dominant zones in each solution. Linear variation for surface tension of the solution is not exhibited. It was confirmed that the two components with different ratios were not added simply. Thus, the varied surface tension is associated with the change in microscopic interaction in the mixed solution.

Figure 2 presents the Raman spectra of the CN stretching band and OH stretching band with different mole ratios at room temperature. With the addition of acetonitrile, the stretching vibration peak of CN shifts to high wavenumbers. This is mainly due to the strong hydrogen bonding between the N atoms of acetonitrile and the H atoms of H_2_O, and the number of hydrogen bonds in the solution increases with the increasing content of acetonitrile, and hydrogen-bonding interaction is strengthened in the range of low concentration as the molar ratio does not in excess of 1. Thus, the CN stretching bond length is elongated so that the vibration force constant decreases, leading to the movement of vibration peak to high wavenumbers. Besides, in the pure water system, the status of water molecules is divided into three categories, including the free water molecules, partially hydrogen-bonded water that forms a hydrogen bond between a water molecule and one or more molecules around it, and a tetrahedral hydrogen bond structure formed with a water molecule surrounded by four water molecules [24, 25, 40]. On the OH stretching band, the shoulder peak around 3200 cm^−1^ is the fully hydrogen-bonded Raman peak of the tetrahedral water structure, and meantime, the peak around 3400 cm^−1^ is the partially hydrogen-bonded peak between the water molecules in the solution [24]. It was found that the fully hydrogen-bonded peak position did not change significantly, whereas the intensity decreased with the addition of acetonitrile to the water solution. Otherwise, the partially hydrogen-bonded water peak intended to move towards high wavenumbers and the relative peak intensities increased gradually. The fact that mainly governs the fully hydrogen-bonded structure broke and the partially hydrogen-bonded structure increased due to the increase in acetonitrile. In addition, the addition of acetonitrile reduced the polarity of water. The electron cloud of the OH bond in the water was toward the O atom originally and then moved to the side of the H atom. The electron cloud density deviated from the center of the two atoms, resulting in the reduction of vibration force constant and the increase of vibration frequency. Hence, the red-shift occurred in the OH bond. As so, the electrons in H were attracted to the N side of the acetonitrile, and the N atoms in the water moved toward the side of H because of H atoms being attracted by hydrogen bonding, so the length of the OH bond reduced and achieved the balance of electron density.

**Figure 3:**
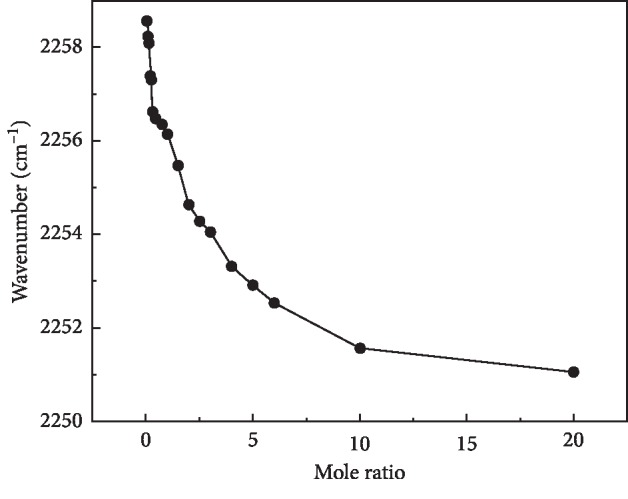
Raman shift of C≡N bonding in acetonitrile aqueous solution with a variety of mole ratios.

**Figure 4:**
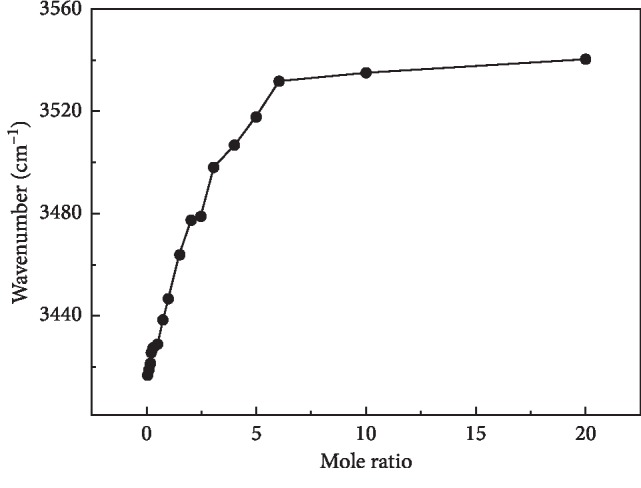
Raman shift of OH bonding in acetonitrile aqueous solution with different mole ratios.

Investigation on varied trends on the Raman shifts for CN and OH bonds based on the interaction between acetonitrile and water is carried out and plotted in Figures 3 and 4. Due to the increased content of water, the characterized peak of CN in the solution gradually shifts to high wavenumbers, as shown in Figure 3. In addition, Figure 4 shows the Raman frequency shift of the OH stretching band. It was observed that the addition of acetonitrile shifts the peak of the OH stretching band to high wavenumbers. Compared with Figures 3 and 4, the blue shift of the OH stretching band is significant, which is higher than that of the CN bond. It is deduced that the addition of acetonitrile reduces the overall ordering structure of water in the solution and decreases the average number of hydrogen bonds in water. In the process of acetonitrile molecules competing with water molecules for the water molecules around them under the hydrogen bonds formed in a solution, remarkable advantages were exhibited for acetonitrile molecules. Therefore, the strong hydrogen bond between the water molecule and acetonitrile was formed. As a consequence, the stable tetrahedral hydrogen-bonded network structure was broken, and furthermore, it results in the increasing partial hydrogen-bonded water. Hence, the surface tension drops continuously in the rich water area in Figure 1. As shown in Figure 3, the Raman shift of the OH bond moved significantly with the addition of acetonitrile and the variation gradient is relatively large when the acetonitrile content is in the low concentration region. However, the Raman shifting slows and the changing gradient reduces when the content of acetonitrile is in the high region, mainly by having the gradual slow broken degree of the hydrogen-bonded structure between water molecules with the increase in acetonitrile. So the amount of tetrahedral hydrogen-bonded structure was reduced, and the partially hydrogen-bonded structure was formed, in which the microstructure of the binary solution system changed greatly. As shown in Figure 4, the Raman shift of CN bond increases with the increasing water content. Accordingly, the hydrogen-bonding interaction between acetonitrile and water resulted in the increase in frequency shift gradient.

It can be still seen from Figures 3 and 4 that the change in frequency of the OH bond and CN bond has the opposite trend simultaneously with the increasing mole ratio. Apparently, both the characterized peaks of two pure solutions move to high wavenumbers with the increase in the other solute. In the water-rich region, the two characterized peaks move to high wavenumbers rapidly, but it is not apparent in the acetonitrile-rich zone due to the addition of acetonitrile, leading to strengthened hydrogen bonding in the mixed solution. So the interaction between molecules changes in the solution. It is apparent that the surface tension changes dramatically with the addition of acetonitrile, as shown in Figure 1. In view of this, the interaction between acetonitrile and water in the solution is weakened, owing to the addition of acetonitrile, determining the properties of solution. In this case, there exists a simple molecular structure in the solution. The change in surface tension was sluggish obviously. It was still proved that acetonitrile has a great influence on the microstructure in water-rich zones of the binary system.

**Figure 5:**
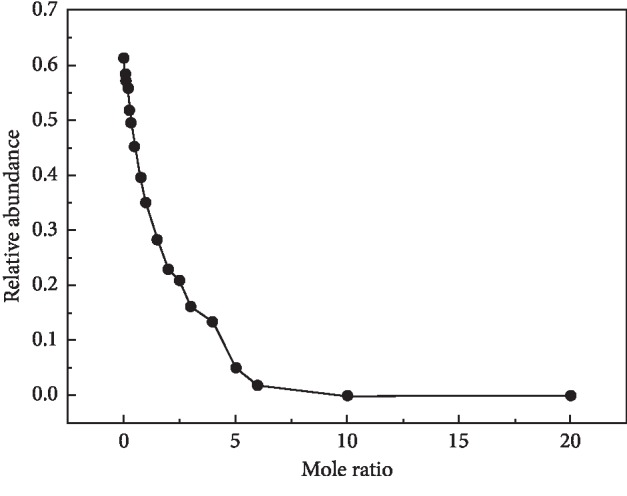
Relative abundance of OH bonding in acetonitrile aqueous solution with different mole ratios.

In order to study the transformation of hydrogen bonding on various structural models in aqueous solution, OH bonds are often divided into multiple subpeaks and, accordingly, assigned into different structures. Risović et al. [37] divided the OH bond into two subbands at 3424.8 cm^−1^ and 3206 cm^−1^ being attributable to non-H-bonded and H-bonded species, respectively. The divided three subpeaks of the OH bond were located at 3241 cm^−1^ for the ordered water structure (ice-like structure) and 3461 cm^−1^ and 3655 cm^−1^ for the disordered structures (defective structures), respectively [36]. Furthermore, there showed the divided five Gaussian components for the OH bond which was assigned to fully or partly hydrogen-bonded water structure [32]. As so, to analyze the hydrogen-bonding structure in the solution in detail, the OH stretching band was fitted into three subpeaks, denoted as PekI, PekII, and PekIII at positions of 3245 cm^−1^, 3550 cm^−1^, and 3420 cm^−1^, individually. To be more specific, PekI is assigned to the tetrahedral hydrogen-bonded water, and PekII and PekIII are assigned to the partially hydrogen-bonded water [24, 27]. The intensity of each peak was obtained and marked as I3245 cm^−1^, I3420 cm^−1^, and I3550 cm^−1^. And the relative abundance of PekI was calculated based on I3245 cm^−1^/(I3245 cm^−1^ + I3420 cm^−1^ + I3550 cm^−1^).  Figure 5 shows the relative abundance of the tetrahedral fully hydrogen-bonded structure and partially hydrogen-bonded structure in solutions with different concentrations. With the increase in the content of acetonitrile, the relative peak intensity decreased. It means that the addition of acetonitrile makes full hydrogen bonding change to partial hydrogen bonding, and destroy the original microstructure of aqueous solution. Thus, the fully hydrogen-bonded water reduced gradually, and the relative content of partially hydrogen-bonded water is up to the level of stability. The water molecule is surrounded by multiple acetonitrile molecules due to the formation of a cluster structure, which is characterized as heterogeneousness and homogeneousness on the micro- and macrolevel respectively, as the molar ratio increases, and furthermore, the content of acetonitrile is much higher than that of water in the solution. The interaction between acetonitrile and water is well explained by the change in relative content of two kinds of hydrogen bonds and the trend in surface tension was anastomosed very well. It was confirmed that the varied surface tension is related to intermolecular interaction in the solution system.

**Figure 6:**
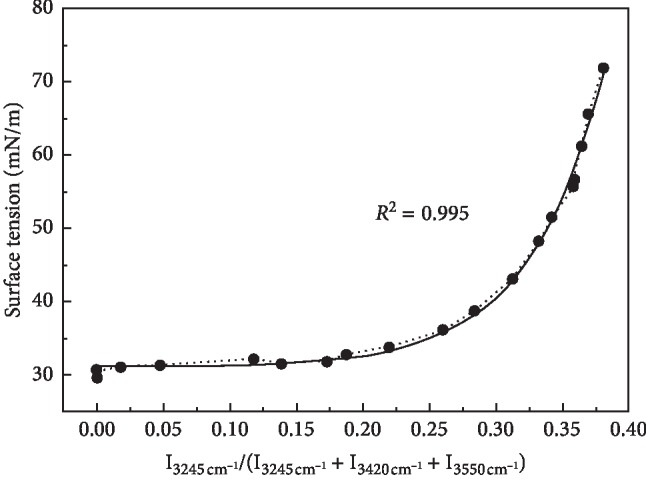
Relationship between surface tension and relative abundance of tetrahedral hydrogen bonds. The fitting curve with the relationship curve between surface tension and relative abundance for the solid line and the dotted line.

The relationship between relative abundance of tetrahedral hydrogen bond structure and surface tension is shown in Figure 6. For understanding the effect of molecular structure on the surface tension deeply, a good exponential function (correlation coefficient square *R*^2^=0.995) was established based on the correlation of surface tension and relative abundance. The surface tension is dominated by acetonitrile solution when relative abundance and water content are in the low region with the few tetrahedral hydrogen bonds. Owing to the increasing water content and the interaction between water and acetonitrile molecules, the molecular structure changes, and meanwhile, the tetrahedral hydrogen bond and the surface tension began to increase. As the high relative abundance is shown, the water content and the tetrahedral hydrogen bond increased continuously. The hydrogen bond between acetonitrile and water is no longer evident in contrast to that of tetrahedral hydrogen bond in water. The distribution of acetonitrile in water solution was considered as nonhomogeneity on the microlevel. To the contrary, the homogeneity on the macrolevel was exhibited. Water molecules have a great influence on the structure of solution. The molecular structure is more stable so that the surface tension increases rapidly. Moreover, the water content continues to increase, and the variation in surface tension ends up at the pure water surface tension. It was deduced that the surface tension was affected by the hydrogen-bond effect in the mixture of binary system.

## 4. Conclusions

The variation in surface tension and hydrogen-bonding effect with the changing molar ratio of acetonitrile aqueous solutions using Raman spectra was investigated. The interesting phenomenon from the experimental results verified that the surface tension of solution with the increasing molar ratio decreased nonlinearly. Moreover, the spectral features of the OH band and CN band were analyzed in the various molar ratios of mixture. Meanwhile, different hydrogen-bonding structures were assigned to Gaussian subpeaks being fitted from the OH stretching. The results showed that the hydrogen-bonding interactions between acetonitrile and water resulted in the change in microstructure in the binary mixture. The transformation from the tetrahedral hydrogen-bonded structure to the partially hydrogen-bonded structure of water was exhibited with the addition of acetonitrile. It was determined that the hydrogen bonding was a reason for reducing the surface tension in the solutions directly. Importantly, a novel way to establish the relationship between microstructure and macroscopic properties of aqueous solutions was provided, and which was the guidance of experiment basis of the spectroscopic applying in aqueous solution.

## Data Availability

The data used to support the findings of this study are available from the corresponding author upon request.
